# Rectal Cancer in the Eye: A Case Report of Orbital Metastasis

**DOI:** 10.7759/cureus.1589

**Published:** 2017-08-21

**Authors:** Mohammed Nabeel, Rehan Farooqi, Mahsa Mohebtash, Rupak Desai, Uvesh Mansuri, Smit Patel, Jinal Patel, Vinshi Naz Khan

**Affiliations:** 1 Department of Internal Medicine, Medstar Union Memorial Hospital; 2 Chief of Medical Oncology and Hematology, Medstar Union Memorial Hospital; 3 Research Coordinator, Atlanta Veterans Affairs Medical Center; 4 Public Health, Icahn School of Medicine at Mount Sinai; 5 Department of Neurology, University of Connecticut Health Center; 6 Department of Internal Medicine, Winthrop University Hospital

**Keywords:** rectal cancer, metastasis, chemotherapy, radiation therapy, orbit

## Abstract

Orbital metastasis from colorectal cancer is extremely rare. In this case report, we describe a 48-year-old woman who presented with recurrent severe headaches and new onset constipation with no known history of cancer. After vigilant workup, imaging, and biopsies, she was diagnosed with orbital metastasis from a primary rectal carcinoma. She was started on chemotherapy and radiation therapy. Her chemotherapy regimen consisted of FLOX (leucovorin + fluorouracil + oxaliplatin), along with panitumumab, which she tolerated well. She received chemotherapy for seven months before she lost her battle with cancer.

## Introduction

Colorectal cancer (CRC) is the second leading cause of cancer-related deaths in the United States in males and females combined [1]. Around 20% of patients with CRC have distant metastases at the time of diagnosis with an involvement of the liver, lungs, peritoneum, or bone [2-4]. Orbital metastasis from colorectal cancer is rare with only a handful of cases in the literature [5]. Metastatic orbital lesions have been estimated to account for 1% to 13% of all orbital tumors with prevalence estimated to range from 2% to 4.7% [5]. 

## Case presentation

A 48-year-old African American female with history significant for hypertension presented to the emergency room with recurrent severe (9/10) bifrontal throbbing headaches for the past few months. These headaches would be exacerbated by strong odors and perfumes and were only minimally refractive to pain medications. Her primary care physician started her on topiramate in an attempt to control these headaches, which were thought to be migrainous. However, this treatment was not effective.

When her symptoms did not improve, she presented to the emergency room with a severe headache. The nature of her headache was similar to previous headaches. However, this time her headache was associated with right eyelid swelling, blurry vision, and black floaters. She denied diplopia, lacrimation, rhinorrhea, ocular trauma, pain, or any discharge. Aside from a headache, she also complained of constipation and rectal pressure. She endorsed nausea and non-bloody, nonbilious emesis. She denied any abdominal pain and weight or dietary changes. Her last bowel movement was around 10 days prior to admission, but she was able to pass flatus. She further denied any fever, chills, or night sweats. 

**Figure 1:**
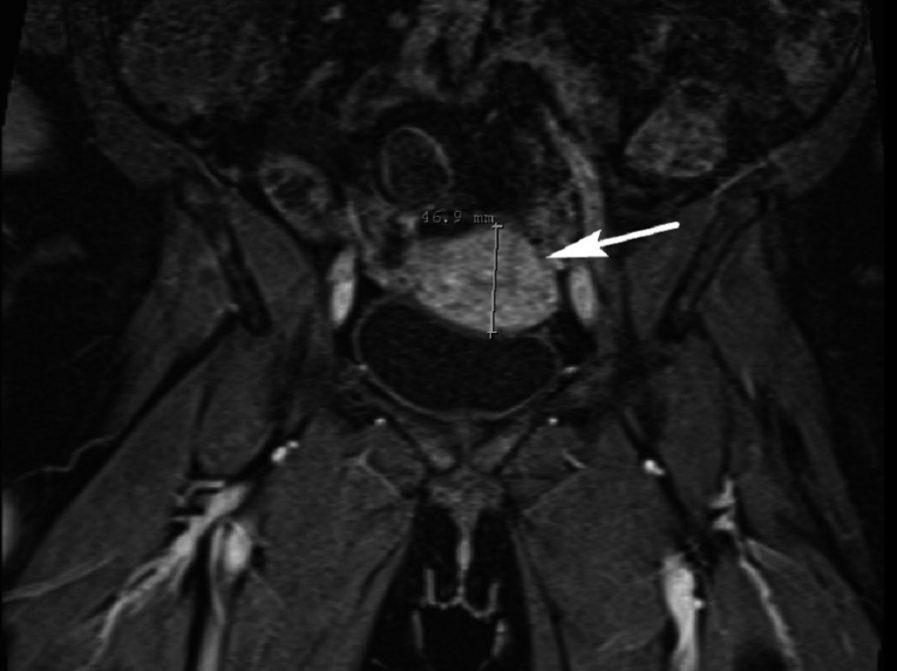
Abdominal magnetic resonance imaging (MRI) Abdominal MRI shows a 4.5 cm concentric apple core tumor (arrow) in the wall of the proximal to mid-rectum region with only a small residual patent lumen present.

**Figure 2:**
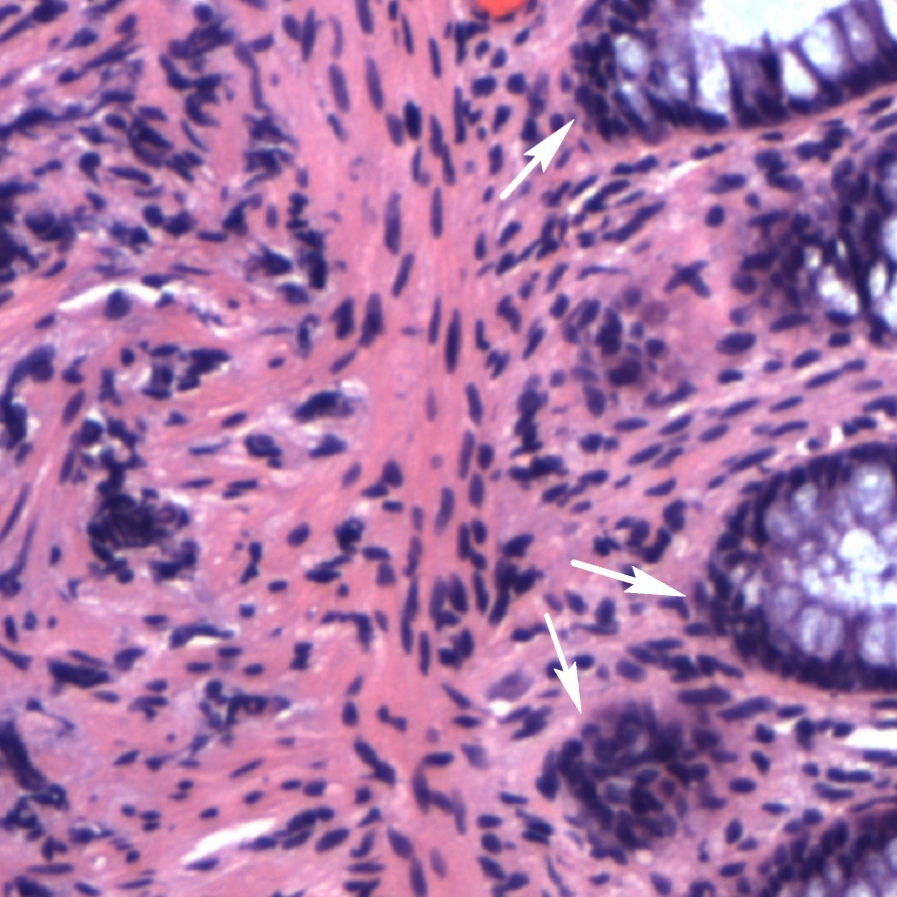
Histology from a rectal mass biopsy Histology from a rectal mass biopsy shows a poorly differentiated carcinoma involving submucosa and muscularis mucosa (arrows).

**Figure 3:**
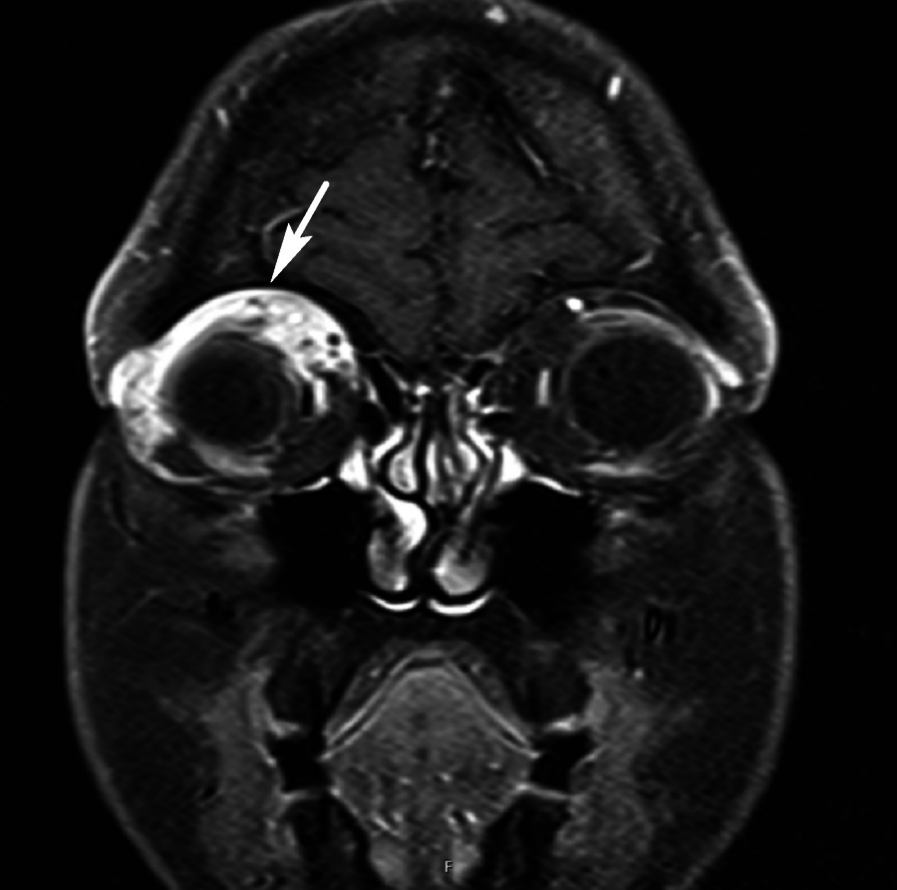
MRI of the head An infiltrative process is noted on MRI of the head in the right orbit (arrow) involving the adjacent muscles and sparing the optic nerve, hypervascular in nature MRI: magnetic resonance imaging

**Figure 4:**
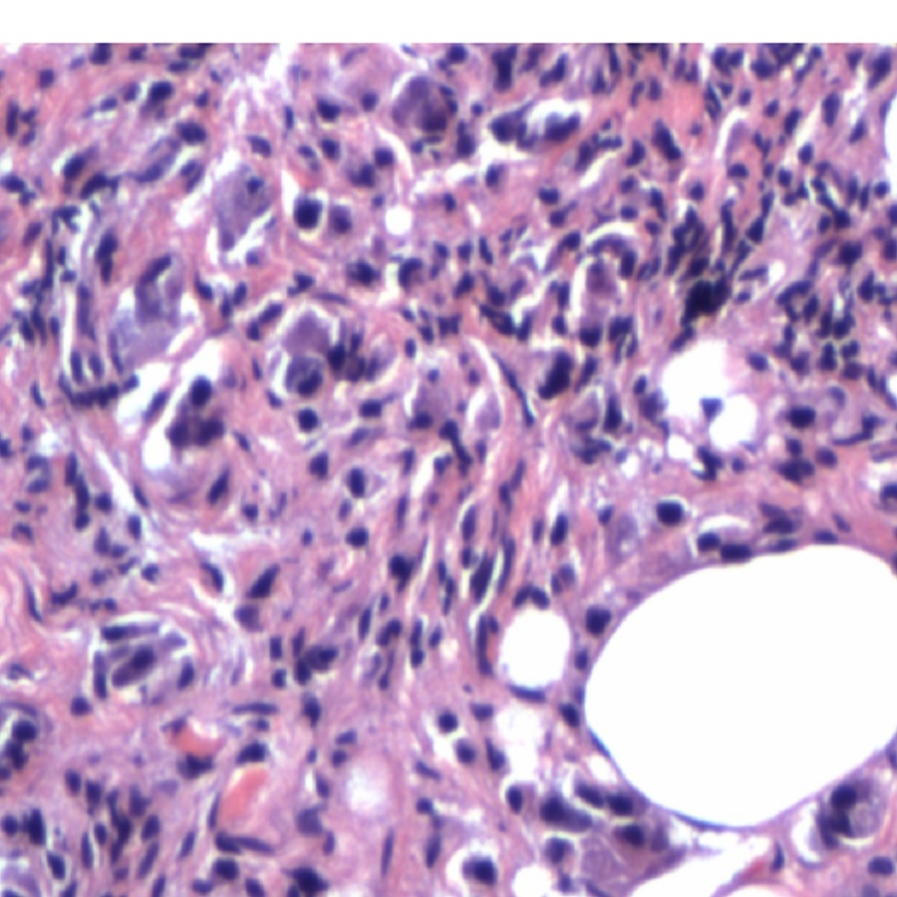
Histology from the right orbital mass biopsy Histology of the soft tissue of the right eye orbit (excision) infiltrating carcinoma. The appearance and immunoprofile of the specimen is similar to that of the rectal lesion, suggesting that the orbital tumor represents a metastasis.

Pertinent physical exam findings included marked ptosis of the right eye with mild proptosis, lid edema, mild conjunctival erythema, full visual fields, and visual acuity of 20/20 bilaterally. Her abdomen was soft, non-distended, and non-tender with normoactive bowel sounds. She had good rectal tone. She was found to have a mass of less than 1 cm in diameter around 8 cm from the anal verge. Computed tomography (CT) of the abdomen and pelvis visualized an obstruction around the site of an annular mass. Magnetic resonance imaging (MRI) of the abdomen confirmed an apple core lesion in the proximal and mid-rectum concerning for malignancy (Figure 1). Colonoscopy was performed, cold biopsies were taken, and the final pathology report was positive for poorly differentiated adenocarcinoma (Figure 2). A whole body CT showed innumerable sclerotic/blastic osseous lesions involving the spine, vertebral bodies, pelvic bones, and proximal femora consistent with diffuse bony metastases. With regard to her headaches, both head CT and orbital MRI were indicative of infiltration of the subcutaneous fat involving the right eyelid (Figure 3). Following an orbital mass biopsy and the pathology report consistent with rectal adenocarcinoma metastasis (Figure 4), palliative treatment was started with 5-fluorouracil and radiation therapy.

Her hospital course was complicated by the development of disseminated intravascular coagulopathy (DIC), most likely as a result of her cancer, but she remained hemodynamically stable. She was started on standard chemotherapy treatment (FOLFOX + prednisone). A week later, upon discharge, oxaliplatin was added to her treatment regimen. Within a month, she underwent a bone scan and a head and chest CT, which showed the development of new right pleural effusions and no change in the size of the rectal mass, consistent with worsening widespread bony metastases. After therapeutic thoracentesis, the effusion was determined to be exudative and compatible with malignancy. Panitumumab was added six weeks from the initiation of chemotherapy and radiation therapy. She received radiation therapy for a total of seven weeks, and after completing her radiation therapy, she was maintained on chemotherapy alone with fluorouracil, leucovorin, and oxaliplatin (FLOX) and panitumumab. Approximately seven months after the initiation of chemotherapy and radiation therapy, our patient lost her battle with cancer. This outcome was inevitable given the poor prognosis of Stage IV rectal carcinoma.

## Discussion

Approximately 20% of patients with colorectal cancer have distant metastases at diagnosis, and 30% develop metastases during their course of the disease [2-3]. The most common cause of ocular malignancy is a metastasis. The most common primary cancers with orbital metastasis include breast, lung, melanoma, and prostate cancers [6]. Gastrointestinal tract cancers account for very few ocular metastasis cases. There are only a handful of such cases in the literature to date. Khwaja, et al. reported a literature review of colorectal cancer with ocular metastases totaling eight patients, including their own [7]. Seven of the eight patients had a known history of colorectal cancer. Only one out of the eight patients, similar to our case, was diagnosed with a primary malignancy of colorectal cancer after the onset of visual disturbance. Also, only one of the eight patients had no widespread metastases. The mean age was 50 years and the mean survival time of the six patients who expired was approximately 10 months. One patient survived 32 months, raising the reported mean survival time. The remaining five patients expired at 16 months, nine months, four months, two months, and one month from their initial presentation, thereby demonstrating extremely high mortality. To our knowledge, our patient case is only the second case in the United States and demonstrates where tumor emboli from the rectum reach the orbit, metastasizing along the way. 

Most orbital metastases present in patients with an established diagnosis of cancer, along with widespread systemic involvement [5]. The typical manifestation of orbital metastases includes diplopia (48%), proptosis (26%), pain (19%), decreased vision (16%), ptosis (10%), or mass, consistent with symptoms reported by our patient [5].

The pathophysiology of metastases is still not well understood. Some have speculated that rectal cancer has easier access to the orbit compared to colon cancer. Rectal cancer metastasizes with tumor emboli traveling through the middle and inferior hemorrhoidal veins to the inferior vena cava, then via the pulmonary circulation to the carotid arteries, and later into the ophthalmic artery [8]. As a result, pulmonary metastasis is common. The other possible route is when rectal cancer seeds into Batson’s venous plexus and reaches the cranial venous sinuses leading up to the ophthalmic vein [9]. In this route, vertebral metastasis is more common. Our patient had evidence of bony metastasis on CT with innumerable sclerotic/blastic osseous lesions involving the spine, vertebral bodies, and pelvic bones.

Orbital metastasis demonstrates poor prognosis in the literature. In one case series of 26 patients, the median survival of patients with metastasis to the anterior segment of the eye was 5.4 months compared to 7.2 months with metastasis confined to the posterior segment and 15.6 months with orbital involvement [10]. To date, treatment is primarily palliative care with the mainstay being external beam radiotherapy combined with chemotherapy related to maintaining the quality of life. Radiotherapy is administered to control tumor growth, preserve visual function, decrease proptosis and, in the process, provide patient comfort [5]. With more such cases being reported in the literature, we can better understand the natural course of the disease and, thereby, develop improved therapies.

## Conclusions

There are uncommon presentations of common diseases as depicted by our case study. It is important to obtain a detailed history, perform a thorough physical exam, and consider further diagnostic imaging if deemed necessary. It is also extremely important to establish the diagnosis early to allow initiation of chemotherapy radiation therapy as soon as possible to improve the quality of life. We believe with more such cases being reported in the literature, there would also be a need for studies and trials to evaluate standard therapy and further novel therapies in the future.
